# Creatine monohydrate supplementation for older adults and clinical populations

**DOI:** 10.1080/15502783.2025.2534130

**Published:** 2025-07-17

**Authors:** Darren G. Candow, Sergej M. Ostojic, Philip D. Chilibeck, Igor Longobardi, Bruno Gualano, Mark A. Tarnopolsky, Theo Wallimann, Terence Moriarty, Richard B. Kreider, Scott C. Forbes, Uwe Schlattner, Jose Antonio

**Affiliations:** a University of Regina, Faculty of Kinesiology and Health Studies, Regina SK, Canada; b University of Agder, Department of Nutrition and Public Health, Kristiansand, Norway; c University of Saskatchewan, College of Kinesiology, Saskatoon, SK, Canada; d Applied Physiology and Nutrition Research Group – School of Physical Education and Sport and Faculdade de Medicina FMUSP, Universidade de Sao Paulo, Sao Paulo, SP, Brazil; e Universidade de Sao Paulo, Center of Lifestyle Medicine, Faculdade de Medicina FMUSP, So Paulo, SP, Brazil; f Department of Pediatrics, McMaster University Children’s Hospital, Hamilton, ON, Canada; g Department of Biology, ETH Zurich, Switzerland; h University of Northern Iowa, Department of Kinesiology and Athletic Training, IA, United States; i Texas A&M University, Department of Kinesiology and Sports Management, College Station TX, USA; j Brandon University, Department of Physical Education Studies, Brandon MB, Canada; k University Grenoble Alpes, Inserm U1055, LBFA, Grenoble, France; l Nova Southeastern University, Department of Health and Human Performance, Davie FL, USA

**Keywords:** Muscle, strength, bone, cognition, memory

## Abstract

**Background:**

The biological process of aging is typically associated with a decrease in muscle quantity, muscle performance (primarily strength), bone mass and architecture, functionality and neurological/cognitive function. From a healthy aging perspective, interventions that have the potential to overcome or attenuate these decrements are clinically relevant.

**Methods:**

We conducted a narrative review on the efficacy of creatine monohydrate supplementation (CrM) in older adults.

**Results:**

Accumulating research shows that CrM, primarily when combined with exercise training, is safe and has beneficial effects on measures of whole-body lean body mass, regional muscle size, muscle strength, bone area and thickness, functional ability, glucose kinetics, cognition and memory.

**Conclusion:**

CrM has multiple benefits in older adults and may have application for treating age-related sarcopenia, osteoporosis, frailty, and those with metabolic and neuromuscular disorders.

## 1. Introduction

Creatine (*N*-(aminoiminomethyl)-N-methyl glycine) is a naturally occurring compound that when combined with phosphate provides energy for cellular metabolism. The daily creatine need is about 2–4 grams/day [1,2]. About half of the daily need for creatine is endogenously synthesized from reactions involving the amino acids arginine, glycine, methionine and stored mostly as free creatine (Cr) or phosphocreatine (PCr) in skeletal muscle [1,3,4]. Specifically, creatine is synthesized from arginine and glycine by the enzyme L-arginine: glycine amidinotransferase (AGAT) to guanidinoacetate (GAA). GAA is then methylated by guanidinoacetate N-methyltransferase (GAMT) with S-adenosyl methionine (SAMe) to form creatine [5]. AGAT is found in the kidney, liver, pancreas, and some areas within the brain, while GAA is mostly formed in the kidney and converted by GMAT to creatine in the liver [6–8]. The remaining daily need for creatine must be obtained from foods containing creatine (e.g. meat and seafood contain about 2–5 grams/kg of creatine) and/or through creatine monohydrate supplementation (CrM). The absolute oral bioavailability of creatine has not been established (due to lack of intravenous data; Persky et al. [9]) but it is estimated to be close to 100% [1]. Creatine in circulation is then taken up into various tissues via tissue-specific creatine transporters (e.g. CRT1 or SLC6A8) and creatine kinases [1,10–13]. The primary role of creatine is to bind with inorganic phosphate (Pi) in the cell to form PCr. PCr is degraded into Pi and Cr to provide energy to resynthesize adenosine triphosphate (ATP) for cellular metabolism [2]. Creatine also plays a critical role in translocating energy-related intermediates from the electron transport system in the mitochondria to the cytosol [10,11]. The availability of PCr is an important energy source for maintaining ATP levels, particularly during metabolically stressful conditions like intense exercise, periods of injury or illness, and some metabolic diseases.

The total creatine pool (Cr + PCr) is about 120–130 mmol/kg of dry muscle mass for an individual who consumes a diet containing meat and seafood [14]. Individuals with AGAT or GMAT enzyme deficiencies and/or creatine transporter deficits have difficulty synthesizing and/or storing creatine. This leads to low PCr levels in tissues for metabolism. Muscle creatine levels are typically lower in vegetarians [15–17] and the elderly who may not consume as much meat or seafood in their diet due to difficulty digesting these food products [18,19]. Dietary supplementation of CrM (e.g. 0.3 grams/kg/day for 5–7 days and 0.05 to 0.15 grams/kg/day thereafter) increases blood, muscle, and tissue levels of creatine and PCr by 20–40% [20–23]. Older individuals with low dietary creatine intake ( <0.95 grams/day) have poorer cognitive function compared to those consuming more dietary creatine ( >0.95 grams/day) [24]. Additionally, analysis of dietary creatine intake among 1,500 adults ≥65 years revealed that 70% of this cohort consumed less than the recommended amounts of creatine in their diet ( <0.95 grams per day), and low dietary creatine intake was associated with a greater risk of angina pectoris and liver conditions compared to those consuming ≥1.0 grams/day of dietary creatine [25]. However, these recommendations should be interpreted with some caution as dietary creatine intake was estimated using recall strategies (i.e. food records) which have high variability due to self-reporting, memory and honesty.

## 2. Creatine kinase system

In order to understand the pleiotropic functions of creatine, the most prominent soluble organic compound in the human body besides water, and its high-energy counterpart PCr, it is imperative to understand how the CK-system works within the framework of cellular energy metabolism and general physiology [26–30].

ATP serves as the universal energy currency in all living cells. ATP is an organic compound with three linearly bound phosphate groups and its hydrolysis, catalyzed by ATPases, releases chemical energy that is crucial for various biological processes, including anabolism, cell growth, cell motility, muscle contraction and relaxation, nerve function, as well as ion and metabolite transport. The hydrolysis of Mg-ATP by cellular ATPases generates adenosine diphosphate (ADP) and inorganic phosphate (P_i_) plus a proton *(H*^*+*^):

$$\mathrm{Mg}-\mathrm{AT}P^{2-}\left(\mathrm{ATPase}\right)\;\Rightarrow \mathrm{Mg}-\mathrm{AD}P^{1-}+{P_{i}}^{2-}+H^{+}$$

However, it would not be a wise strategy for cells and organs, which need and utilize large amounts of energy in a short period of time, to simply accumulate high concentrations of ATP as an energy reserve. Since hydrolysis of such vast amounts of ATP and concomitant accumulation of ADP plus H^+^ would eventually bring cellular energy production and metabolism to a halt, due to product inhibition of the ATPases by ADP, P_i_^2−^ and H^+^, as well as further interference of ADP with general metabolism and cellular acidification by H^+^ [26]. This generally poses a significant challenge for organs and cells with high ATP turnover rates, such as skeletal, cardiac and smooth muscles, brain, kidney, nerve cells, immune cells, spermatozoa and others [11,31].

Astonishingly, some 650 million years ago, at the dawn of metazoan branching [32], nature developed a powerful energy back-up system in the form of the enzyme creatine kinase (CK) and its substrates: Cr and PCr. The CK-catalyzed reaction is fully reversible, and its final output will depend on the local concentrations of substrates and products:

$$\mathrm{PC}r^{2-}+\;\mathrm{Mg}-\mathrm{AD}P^{-}+H^{+}⇐\left(\mathrm{CK}\right)\Rightarrow \mathrm{Mg}-\mathrm{AT}P^{2-}+\;\mathrm{Cr}$$

In vertebrates, four evolutionarily related CK genes are responsible for the production of five different but homologous CK isoforms, two mitochondrial MtCK isoforms forming octamers, represented by ubiquitous u-MtCK (CKMT1) and sarcomeric s-MtCK (CKMT2), and three cytosolic CK dimers, represented by muscle-type MM-CK dimers, brain-type BB-CK dimers and a cardiac-specific MB-CK hybrid isoform [26,33]. As indicated by their names, these CK isoforms are differentially expressed in different tissues and cells, and in addition, they are specifically localized within cells, either at sites of ATP production or sites of ATP consumption.

**Figure 1.**
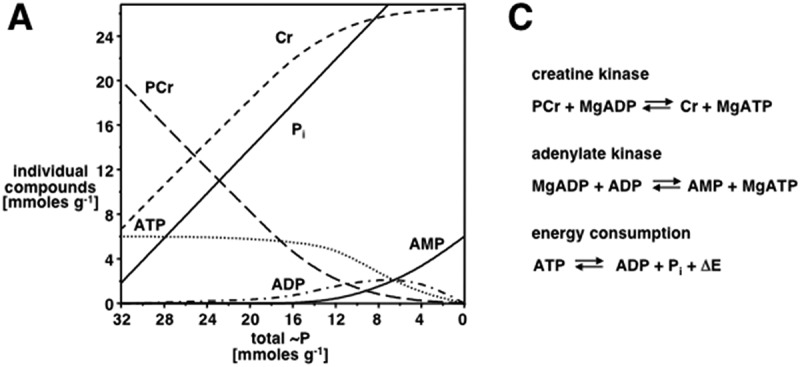
The energy buffer function of the CK-system: PCr as temporal energy reservoir A) model calculations for changes in pool sizes of energy-related metabolites during cell stimulation until exhaustion. Global cellular concentration changes in phospho-creatine [PCr] and adenine nucleotides ([ATP], [ADP] and [AMP]), inorganic phosphate [Pi], and creatine [Cr] are calculated from the reactions of creatine kinase (CK), adenylate kinase and a generalized ATPase. The decreasing “high-energy” phosphates (total ˜P), represented by PCr and ATP, correspond to a transition from rest to high workload and finally exhaustion. At rest, [PCr] and [ATP] are high, while [ADP] is very low and [AMP] is virtually non-measurable. Note that with high-energy phosphate consumption, [ATP] remains constant until more than 80% of the PCr pool is consumed, and only then do [ADP] and later [AMP] start to rise. [Cr] rises proportionally with the decrease of [PCr], and [Pi] increases linearly with the decrease of [PCr] and [adenine nucleotides] combined. Note that during cellular work with increasing PCr depletion, the concentration of Cr, Pi and ADP are increasing and all three are stimulating oxidative phosphorylation for production of ATP by mitochondria, while rising Pi additionally stimulates glycogenolysis and glycolysis to even generate more ATP. Thus, the CK-system serves as an energy buffer to keep [ATP] and the ATP/ADP ratio high and thus guarantees for a high subcellular phosphorylation potential ([Atp]/[ADP]*[pi]), near the various ATPases, where CK is specifically colocalized with- and functionally coupled to- the latter (modified from ref [46]).

In those cells and organs mentioned above, the CK-system with PCr as a relatively large cellular energy reservoir (20–40 mM of PCr), depending on cell type, works as an immediately available energy buffer to regenerate cellular ATP from ADP and to keep the ATP concentration as well as the ATP/ADP ratio at a constant high level. At the same time, products of the ATPase reaction (ADP, H^+^) that would otherwise interfere with metabolism and acidify the cytoplasm, respectively, are removed by the CK reaction. Most importantly, by increasing the Gibbs free energy change of ATP hydrolysis: *∆G = ∆G*_*o*_ **+** RT_*_ ln[ADP]_*_[Pi]**/**[ATP] [34,35], the CK- system increases the energy efficiency of all cells and organs that use ATP. Since the free-energy change (∆G) of ATP hydrolysis directly depends on the local phosphorylation potential (ATP/ADP_*_P_i_) and thus on the local ATP/ADP ratio, more energy is gained per ATP that is hydrolyzed in a cell, if ATPases are subcellularly functionally coupled to CK, which removes ADP. These functionally coupled microcompartments between CK and ATPases make it possible that optimal PCr/ATP and ATP/ADP ratios are maintained near these ATPases, thus optimizing their function (see Figure 1). At these sites of ATP consumption, due to the physiologically given local substrate concentrations with constant PCr supply, the CK reaction is driven toward net ATP synthesis and thus will proceed almost entirely in the direction of regenerating ATP from PCr, as follows:

$$\mathrm{PC}r^{2-}+\mathrm{Mg}-\mathrm{AD}P^{-}+H^{+}\left(\mathrm{CK}\right)\Rightarrow \mathrm{Mg}-\mathrm{AT}P^{2-}+\mathrm{Cr}$$

PCr serves as an energy store for rapid local regeneration of ATP and thus functions as an energy buffer for rapid local regeneration of ATP, e.g. for muscle contraction (see Figure 1). In terms of maximal rates, CK can generate ATP one order of magnitude faster than oxidative phosphorylation and also much faster than glycolysis [36,37]. The energy buffer function of the CK-system relies on the subcellular micro-compartmentation and colocalization of cytosolic CK isoenzymes with various ATPases that are supported and reinforced in their physiological function by functional coupling with CK. This has been extensively studied with the myofibrillar Mg^2+^-activated actomyosin ATPase for muscle contraction, where MM-CK is specifically localized at the sarcomeric M-band in between the actomyosin overlap zones [38]. There, the M-band MM-CK was shown to be sufficient to regenerate *in situ* the ATP hydrolyzed during muscle contraction [13,33]. In support of this, transgenic MM-CK knockout mice show significant alterations in myofibrillar function [39]. Another important functionally coupled micro-compartment is that of MM-CK with the SR-Ca ^2+^-pump ATPase [40–42]. The SR Ca^2+^-uptake operates with a free energy change of ΔG_Ca_^2+^_−transport_ of approximately +51 kJ/mol, and the Gibbs free energy change of ATP hydrolysis at physiological concentrations of ATP (5–8 mM), free ADP (0.02–0.04 mM), and Pi (5–10 mM) in resting muscle may be estimated to be approximately −55 kJ/mol (see review [43]). Thus, the Gibbs free energy provided by physiological concentrations of ATP only slightly exceeds the ΔG required for the thermodynamically unfavorable Ca^2+^-uptake, and maintenance of a high local ATP:ADP ratio is required for efficient sequestration of Ca^2+^ into the SR lumen. Inhibition of SR-bound CK or of CK associated with other ion pumps, e.g. by oxidative damage via ROS, as seen in many neuromuscular and neurodegenerative diseases [44], including mitochondrial myopathies [45], therefore would decrease ΔG _ATP_, and so limit the thermodynamic driving force of the SR-Ca ^2+^ pump or other ion pumps, causing a decrease in contractile reserve. A chronic reduction in cellular energy status can lead to pathological Ca^2 +^ overload and exacerbate the generation of reactive oxygen species (ROS), which are characteristic of such diseases [44]. This is where sarcoplasmic reticulum (SR)-associated creatine kinase (CK) becomes crucial, as it helps to maintain a high local ATP/ADP ratio near the SR Ca^2 +^ pump, thereby ensuring optimal function of this energy-demanding Ca^2+^-pump (for review see [46]). Interestingly, the most striking phenotype in transgenic mice, lacking both cytosolic MM-CK and MtCK, is that these mice have problems with intracellular Ca^2+^-handling and muscle relaxation [47]. Similarly, CK is also specifically associated with the Na^+^/K^+^-ATPase and with the ATP-sensitive K^+^_ATP_ channel, where it optimizes the energetics of the Na^+^/K^+^-pump [48] and regulates the sub-membrane ATP/ADP ratio, thus permitting opening of the K^+^_ATP_ channel even if bulk cytosolic ATP concentration remains high [49]. In transgenic MM-CK k.o. mice, regulation of the K^+^_ATP_ channels by PCr and concomitant signal delivery to the channels is disrupted, resulting in a phenotype with increased electrical vulnerability, for example in cardiomyocytes [50].

Besides this buffer function, the CK-system, with PCr and Cr, works as a continuous energy transport or shuttle system between ATP production sites (mitochondria and glycolysis) and ATP consumption sites, where various ATPases are at work, e.g. for muscle contraction and relaxation by sequestration of free Ca^2+^ and for maintenance of membrane potentials.

**Figure 2.**
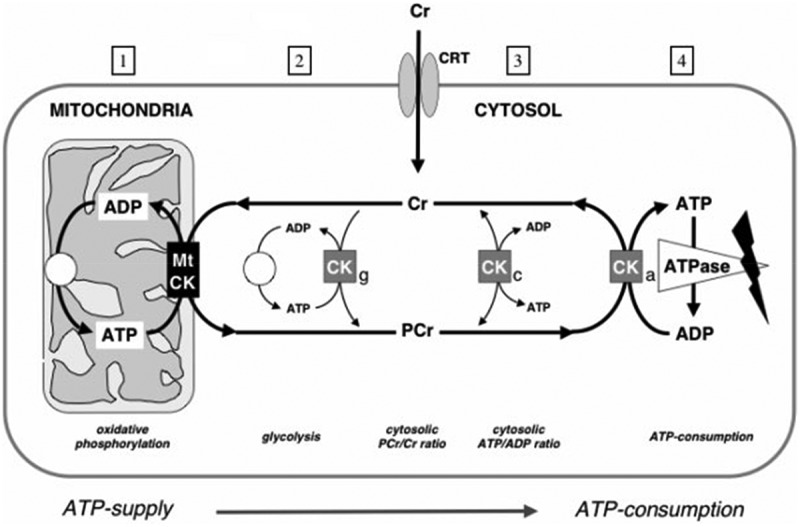
The CK–phosphocreatine shuttle: PCr/Cr for intra-cellular energy transport creatine (Cr), either synthesized in the body or taken up from alimentary sources, e.g. meat and fish, is transported by a specific creatine transporter (CRT) into muscle and other cells that require high and fluctuating energy use. Imported Cr is charged to the high-energy compound phosphocreatine (PCr) by the action either of soluble cytosolic CK (CKc), by CK coupled to glycolysis (CKg), or by mitochondrial CK (MtCK). According to the CK equilibrium reaction, in a resting cell, this results in approximately 2/3 of PCr and 1/3 of Cr and in a very high ATP/ADP ratio of more than 100:1. Some fraction of cytosolic CK is specifically associated (CKa) with ATP-consuming processes (ATPases), such as the myofibrillar actomyosin ATPase, the SR Ca^2+^-ATPase, the plasma membrane Na^+^/K^+^-ATPase, the ATP-gated K^+^-channel, or with ATP-dependent cell signaling. There, CK regenerates directly *in situ* all the ATP utilized by these processes, drawing from the large PCr pool. This represents the ATP-consuming end of the PCr shuttle.

The PCr energy shuttle concept is based on the specific subcellular localization of CK isoenzymes at these various sites [12,51] and on the high cytosolic concentrations of free creatine (5–10 mM) and phosphocreatine (20–45 mM) compared to ADP (0.02–0.04 mM) and ATP (3–5 mM) [27,28]. (see Figure 2).

In addition, CK is also associated to glycolytic enzyme complexes (CKg), where glycolytically generated ATP is trans-phosphorylated into PCr that is then fed into the large PCr pool. This represents the first of the two ATP-producing sites of the PCr shuttle. The second ATP-producing site resides in mitochondria, where mitochondrial MtCK is specifically located in the intermembrane space of mitochondria. By functional coupling of MtCK to the adenine nucleotide translocator (ANT) of the mitochondrial inner membrane, MtCK preferentially accepts mitochondrially generated ATP and trans-phosphorylates it into PCr, which then leaves the mitochondria. A large cytosolic PCr pool of up to 20–45 mM is built up by CK using ATP from oxidative phosphorylation, as in the heart, or from glycolysis, as in fast-twitch glycolytic skeletal muscle. PCr is then used as an energy reservoir to buffer global cytosolic and local ATP:ADP ratios. In cells that are polarized and/or have very high or localized ATP consumption, the differentially localized CK isoenzymes, together with easily diffusible PCr and Cr, can maintain a high-energy PCr flux or PCr-shuttle between ATP-providing, depicted by (1) and (2) in Figure 2, and ATP-consuming processes, as indicated by (4) in Figure 2). Thus, the energy producing and consuming terminals of the shuttle (see Figure 2), are connected preferentially via PCr and Cr, with no need for ATP to diffuse, e.g. from mitochondria (1) to the sites of ATPases (4), or even more so for ADP to diffuse from the ATPases back to mitochondria. Note that in this PCr-shuttle, MtCK and CKa isoenzymes operate in opposite direction (taken from [46]).

The basics for the PCr energy shuttle have been elaborated first in cardiac and skeletal muscle, based on results obtained with relatively simple experiments using isolated mitochondria from heart [52] and isolated muscle fibers or myofibrils from skeletal muscle and heart [26,31,33,53].

**Figure 3.**
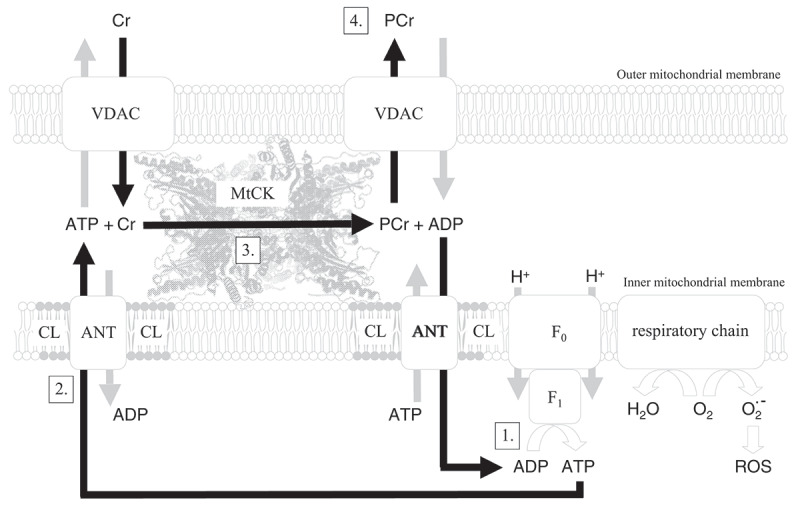
The mitochondrial side of the PCr/Cr-shuttle: production of high-energy PCr by mitochondria. In this scheme, ATP generated by oxidative phosphorylation via the F1-ATPase (1) is transported through the mitochondrial inner membrane (IM) by the adenosine nucleotide transporter (ANT) (2) in exchange for ADP. This ATP is preferentially accepted and trans-phosphorylated into PCr by octameric MtCK in the intermembrane space (3). Under certain circumstances, some of this ATP may also be directly exported through the voltage-dependent anion carrier (VDAC) (at left) [58]. However, it is preferentially PCr that leaves the mitochondrion via VDAC (at right) to which MtCK can directly interact with in a Ca^2+^-dependent fashion. This PCr then feeds into the large cytosolic PCr pool (4). ADP generated from the MtCK transphosphorylation reaction is accepted by ANT and immediately transported back into the matrix (5) to be recharged by the F1-ATPase (1). In contact sites, this substrate channeling allows for a constant supply of substrates and concomitant removal of products at the active sites of MtCK. In cristae, where MtCK is also localized, only ATP/ADP exchange is facilitated through direct channeling to the MtCK active site, while Cr and PCr have to diffuse along the cristae space to reach the VDAC (not shown here, for details, see [55]). Interaction of MtCK with cardiolipin in the mitochondrial inner membrane (IM) surrounding ANT, as well as tight functional coupling of MtCK to ANT leads to saturation of the ANT on the outer side of the IM with ADP, which is transported back into the matrix (5) to be recharged by the F1-ATPase (1), thus efficiently coupling electron transport to ATP generation and at the same time lowering the production of free oxygen radicals (ROS) [57]. On the other hand, the tight functional coupling of ANT to MtCK leads to a saturation of MtCK with ANT-delivered ATP and a locally high ATP:ADP ratio in the vicinity of MtCK, and in combination with cytosolic Cr, entering the intermembrane space via VDAC (at the left), it drives the synthesis by MtCK of PCr from ATP without a loss of its energy content, thus maintaining maximal thermodynamic efficiency for high-energy phosphate synthesis in the form of PCr (4), which then is exported into the cytosol. This explains why Cr stimulates mitochondrial respiration via the action of MtCK [56] (adapted from [46,55]).

At the mitochondrial side of the PCr-shuttle (see Figure 2 on the left), octameric mitochondrial MtCK isoenzyme [54] is specifically localized in the mitochondrial inter-membrane space, that is, between the inner and outer mitochondrial membrane, and is functionally coupled to mitochondrial respiration [55]. By this mechanism, the generation of ATP by oxidative phosphorylation is stimulated by Cr and results in the net production of PCr leaving the mitochondria [56]. At the same time, mitochondrial production of oxygen radicals (ROS) is significantly attenuated by Cr [57]; also see Figure 3. Interestingly, mitochondrial Cr-sensitivity is lost in the D2*mdx* model of Duchenne muscular dystrophy [58]. Functional coupling of MtCK with oxidative phosphorylation [56] helps to minimize the Gibbs free energy required for mitochondrial ATP synthesis: ∆G =∆G_o_ **+**RT_*_ln[ATP] /[ADP]_*_[Pi] [34], by maintaining a high local ADP/ATP ratio in the mitochondrial matrix compartment in the vicinity of the ATP-synthetase and a high local ATP/ADP ratio in the mitochondrial intermembrane compartment in the vicinity of the mitochondrial CK (see Figure 3; and for review see ref. [43]). As a result of this micro-compartmentation within the two mitochondrial compartments, facilitated by the adenosine translocase (ANT) and MtCK, the CK reaction is driven toward net synthesis of PCr due to constant ATP supply by the ATPase and its translocation via ANT, with PCr being released from mitochondria (see Figure 3). Thus, the enzymatic CK reaction by MtCK can be written as follows:

$$\mathrm{Mg}-\mathrm{AT}P^{2-}+Cr^{\;}\left(\mathrm{MtCK}\right)\Rightarrow \mathrm{Mg}-\mathrm{AD}P^{-}+\;\mathrm{PC}r^{2-}+\;H^{+}$$

At cellular sites of glycolysis, a dimeric cytosolic CK isoenzyme (either muscle-type MM-CK or brain-type BB-CK) [59,60] is interacting with specific glycolytic enzymes and trans-phosphorylating glycolytic ATP for the net production of PCr. This functional coupling of ATP-synthesis with CK helps to generally minimize the Gibbs free energy required for ATP synthesis. Similar to the situation in mitochondria, the CK reaction is driven toward net PCr synthesis due to constant glycolytic ATP supply.

PCr generated at both mitochondrial and glycolytic sites is then available to various ATPases at the consuming end of the PCr-shuttle. At these sites, as outlined already above, a fraction of cytosolic CK is specifically associated with these ATP-consuming processes (see Figure 2 on the right) such as the myofibrillar actomyosin ATPase, the SR Ca^2+^-ATPase, the plasma membrane Na^+^/K^+^-ATPase, the ATP-gated K^+^-channel, or with ATP-dependent cell signaling. At all these places, CK regenerates *in situ* the consumed ATP by drawing from the large PCr pool, as discussed above.

Since PCr is present at up to 10 times higher concentrations than ATP, and since Cr and PCr are smaller in size and less charged than ATP and ADP, they diffuse faster within the cells than the two nucleotides [37,61]. The latter are also significantly hindered in their diffusion by interaction with various proteins. For example, in resting muscle cells, freely diffusible ADP that is present at very low concentrations (between 0.02–0.04 mM) while much is bound to F-actin filaments. Therefore, PCr and Cr are generally much better suited for intra-cellular energy shuttling compared to ADP and ATP. This fact is documented best in spermatozoa, where PCr has to travel the long distance from the mitochondrion behind the sperm head to the very distal end of the very long sperm tail [61–63]. The PCr-shuttle, however, is of great relevance not only for spermatozoa, but also for oxidative skeletal and even more so for cardiac muscle that depend on a reliable continuous flow of energy inside myocytes and cardiomyocytes, respectively, from mitochondria to the sites of ATP utilization, e.g. to the myofibrils for oxidative skeletal and cardiac muscle contraction and performance [64–66]. Interestingly, over-expression of the creatine transporter (CRT) in transgenic mice with concomitant augmentation of Cr in these murine hearts protects against ischemia-reperfusion damage [67] and overexpression of MtCK in murine hearts improve functional recovery from and protect against injury following ischemia-reperfusion [68].

The inner workings of the CK-system on the mitochondrial side of the shuttle are depicted in Figure 3 that represents an instructive example of functional coupling of MtCK with oxidative phosphorylation and metabolite channeling through the mitochondrial membranes. MtCK octamers are sandwiched between the inner (IM) and outer OM) mitochondrial membrane in the mitochondrial inter-membrane space (IM). MtCK octamers interact with the adenine nucleotide carrier (ANT) of the IM and the cardiolipin clusters surrounding it [69] and between the voltage-dependent anion carrier (VDAC) of the OM, where MtCK octamers interact with VDAC in a Ca^2+^-dependent manner [70]. Thus, MtCK preferentially accepts the ATP that is produced by oxidative phosphorylation and that is transported through the IM by ANT into the inter-membrane space. There, MtCK trans-phosphorylates this ATP to PCr that is then exported through VDAC into the cytosol (see Figure 3) (for review see [55]).

Finally, considering the metabolic disruptions that occur in aging cells and organs, Cr supplementation is advantageous in rectifying disrupted energy metabolism by replenishing the PCr pool and improving the PCr/ATP energy state [2,71] and by stimulating mitochondrial respiration [56] and also by influencing cell signaling pathways [10]. Cells with a high energy charge are able to better master cellular stress situations, e.g. caused by ischemia and hypoxia [68,72–74] or calcium overload [75], and to tolerate or neutralize cytotoxic substances like beta-amyloid [76] or free oxygen radicals (ROS) [77] that are generated during ischemia-reperfusion [66] or by treatment with anthracycline cancer medication [78–80]. Last but not least, Cr protects mitochondria against Ca^2+^-induced swelling and opening of the so-called transition pore that leads to cell death by apoptosis [81,82].

Thus, CK-system provides numerous cell protective effects against a variety of metabolic and cellular stress situations and over an entire lifespan, is likely contributing to healthy aging, resilience and longevity. Therefore, the notions “a *miserable life without Creatine*” [83] and “*Creatine for Life*” indeed rest upon a solid scientific foundation. Most importantly, the CK-system minimizes the thermodynamic costs of ATP synthesis and at the same time maximizes the thermodynamic efficiency of ATP hydrolysis (ATP utilization), and CK-system facilitates high-energy transport via PCr and Cr shuttling throughout the cells. All together the efficiency and economy of cellular energy metabolism are improved and cell performance is optimized by the CK-system. This positively affects numerous aspects of cellular and organismic life, thus justifying the connotation “*Creatine for Life*.”

## 3. Creatine supplementation and aging muscle

Sarcopenia is characterized by the age-related reductions in muscle performance (primarily muscle strength), lean mass and functional ability [84,85]. Sarcopenia increases the risk of falls, fractures and disability [85]. Mechanistically, contributing factors to sarcopenia include changes in neuromuscular and neurophysiology, muscle fiber composition, muscle protein turnover (including anabolic resistance to dietary protein ingestion), mitochondrial function, vascularization, telomere attrition, genomic stability and increases in low-grade inflammation, oxidative stress and cellular senescence [86,87]. Globally, it is estimated that 200 million older adults (≥65 years of age) will experience sarcopenia by the year 2050 [88]. Therefore, interventions which improve muscle performance, lean mass, muscle growth and functional ability are critical for promoting healthy aging and improving the quality of life for older adults worldwide.

The most potent non-pharmacological intervention for treating sarcopenia is resistance training [89]. In addition to resistance training, there is a growing body of evidence which shows that CrM also provides muscle and functionality benefits to older adults. Several meta-analyses have been performed showing that the combination of CrM (≥5 grams/day) and resistance training improves measures of muscle strength [18,90–92], a key factor in the diagnosis of sarcopenia in older adults. Greater muscle strength is also associated with a reduced risk of falls and frailty [81]. However, the efficacy of CrM for improving upper- vs. lower-body strength in older adults may differ. The most recent meta-analysis involving CrM and resistance training in older adults (*n* = 1063) showed that CrM significantly improved upper-body strength (Standardized mean difference [SMD]: 0.24, 95% CI: 0.05 to −0.43; *p* = 0.02) compared to resistance training alone [90]. CrM consistently improves chest press and/or bench press strength [18,90,91,93–97] compared to resistance training alone which has practical applications for performing basic and instrumental activities of daily living (i.e. lifting, pushing) [89]. Another recent meta-analysis also showed that CrM improves hand-grip strength in older adults (*n* = 325; SMD: 0.23; 95% CI: 0.01 to 0.45, *p* = 0.04) [91]. This is important as hand-grip strength is routinely used as a predictor of health outcomes in older adults (i.e. hospitalization, physical disability); [98,99] and hand-grip strength is positively associated with whole-body strength [92]. In contrast, the effects of CrM on lower-body strength are much less robust. Forbes and Candow [90] failed to find a statistical improvement in lower-body strength from CrM compared to placebo in 663 healthy older adults (SMD: 0.17; 95% CI: −0.03 to 0.38; *p* = 0.09). Davies et al. [91] also found no greater benefits from CrM on lower-body strength in older adults (with and without chronic disease; *n* = 463) (SMD: 0.07; 95% CI: −0.18 to 0.31, *p* = 0.61). These mixed findings in relation to lower-body strength may be due to possible age-related changes in muscle morphology, creatine kinetics and/or the CrM dosing protocol used. There is evidence that lower-body muscle groups are more negatively affected (i.e. reduced muscle strength and power) than upper-body muscle groups, especially at high velocities (i.e. 3.14 rads/second) in healthy older males compared to healthy younger males [100]. Specifically, healthy older men produce ~ 30% less maximal strength (determined by isokinetic peak torque) in the knee flexors and extensors and ankle plantar flexors and ~ 20% less maximal strength in the elbow flexors and extensors compared to maximal strength measures of the same muscle groups in healthy younger men [100]. Similarly, average power output for the knee flexors and ankle plantar flexors (3.14 rads/second) in healthy older men is ~ 50% lower compared to average power output of the same muscle groups in healthy younger men [100]. Potentially, greater lower-body strength deficits may mask the small beneficial effects of CrM (compared to placebo) during a resistance training program in healthy older adults. Furthermore, there is some evidence that older adults have lower PCr stores in the *Vastus lateralis* (Standardized mean difference: −0.53, 95% confidence interval CI: [−0.88 to −0.18]; *p* = 0.003) compared to younger adults [95]. Subsequently, older adults may require higher daily doses of creatine to produce more consistent lower-body strength improvements over time [101]. A 2021 meta-analysis review showed that a CrM loading phase (i.e. 20 grams/day for 5–7 days) followed by a CrM maintenance phase of >5 grams/day produced significant improvements in lower-body strength (SMD: 0.29; 95% CI: 0.04 to 0.54; *p* = 0 0.02) in older adults (*n* = 426). Lower-body muscle strength was not significantly improved (compared to placebo) in those studies that did not incorporate a CrM loading phase (SMD: 0.06; 95% CI: −0.24 to 0.36; *p* = 0.69) [18]. Future research should further prioritize the development of CrM and resistance training strategies which improve lower-body strength in older adults due to its important role in performing activities of daily living (i.e. walking, climbing stairs).

There is some evidence that the combination of CrM and resistance training can improve functional ability in older adults. Three meta-analyses have shown that the combination of CrM and resistance training enhances a measure of functional ability (e.g. sit-to-stand test) in older adults [91,93,96]. Most recently, Davies et el. [91] showed that CrM improved sit-to-stand performance in older adults (*n* = 188; SMD: 0.51; 95% CI: 0.01 to 1.00; *p* = 0.04). Using Bayesian analysis, there was also a 66.7% probability that CrM improved physical function over time.

In addition to muscle strength and functional ability, there is substantial evidence that CrM (≥3 grams/day) and resistance training increases whole-body lean mass (primarily determined using dual energy x-ray absorptiometry) by ~1.2 kg more than resistance training alone in healthy, non-frail older adults [18,91,94–97,102]. Greater lean mass is associated with improved cardiovascular health and lower overall mortality in older adults [103]. There is also supporting evidence that CrM can increase regional (limb) muscle size and density. In a small meta-analysis, Burke et al. [104] showed that CrM (0.1 grams/kg/day) and resistance training (≤1 year) had a very small (effect size: 0.06) beneficial effect on improving muscle size (primarily determined using ultrasound) of the elbow and knee flexor and extensor muscle groups. In a recent study using peripheral quantitative computed tomography, 1 year of CrM (0.1 grams/kg/day) and resistance training significantly improved lower leg muscle density (Δ +0.83 ± 1.15 mg·cm^3^; pP = 0.016) compared with placebo (Δ −0.16 ± 1.56 mg·cm^3^) in healthy older adults. These results may have clinical applications as low muscle density is an independent risk factor for falls in older adults [105]. Specifically, for every mg·cm^3^ decrease in muscle density, there is a 17% increase in the likelihood of reporting a fall in older adults [105].

While the etiology explaining the muscle and functionality benefits in older adults from CrM remains largely unknown, there is evidence (primarily in young populations) that CrM stimulates anabolic and anti-catabolic processes. CrM has been shown to influence cell swelling, myogenic transcription factor and satellite cell activity, muscle and whole-body protein kinetics, growth factors, inflammation and oxidative stress [93,95,106]. However, these mechanisms have not been substantiated in older populations.

In summary, there is accumulating evidence that CrM (≥3 grams/day) combined with resistance training is a viable intervention for improving strength, whole-body lean mass, regional muscle size and density and select measures of functional ability in older adults. Future research should determine whether CrM, with and without exercise training, provides muscle and functionality benefits for those diagnosed with sarcopenia and associated age-related conditions such as osteosarcopenia, frailty and cachexia.

## 4. Creatine supplementation and aging bone, Falls and frailty

CrM during resistance training programs have some potential to improve bone health and prevent falls, but there is lack of evidence that it can improve strength, lean body mass, muscle accretion, or functional ability in those who are classified as frail. The sections below review the potential for creatine to affect bone health, prevent falls, and alleviate frailty.

## 5. Bone health

When creatine is added to cell culture there is an increase in activity of osteoblasts [107], the cells involved in bone formation, and CrM during resistance training programs in older men is effective for reducing bone resorption (i.e. bone catabolism): When given 0.1 grams/kg of CrM on training days (3 days/week for 10 weeks), urinary cross-linked N-telopeptides of type I collagen (a marker of bone resorption) decreased by 27% compared to an increase of 13% for those on placebo [108]. Osteoblasts use PCr to buffer ATP and therefore CrM may increase the energy status of these cells [109]. Increased osteoblast activity results in the release of osteoprotegerin, a protein secreted from osteoblasts which signals an inhibition of osteoclast differentiation (i.e. the cells involved in bone resorption) [110]. These potential effects of CrM on osteoblasts need to be replicated *in vivo* in humans.

A preliminary small study showed CrM was effective for improving bone mineral density at the femoral neck during a one-year resistance training program in postmenopausal women [110]. Women given 0.1 grams/kg/day of CrM lost 1.2% bone mineral density at the femoral neck compared to those on placebo who lost 3.9% [111]. This attenuation in bone mineral density loss from CrM approaches a level of clinical significance, where a 5% difference in bone mineral density results in a 25% difference in fracture rate [112]. This effect however was not replicated in a much larger two-year study from the same research group [113]. The lack of effect of CrM on bone mineral density in older adults has also been confirmed by other research groups [114]. CrM may however be effective for changing the shape or geometric arrangement of bone during resistance training [113,115]. Geometric properties are excellent predictors of bone strength [116]. For example, higher bone cross-sectional area strengthens bone placed under compression [117]. Higher cortical thickness and section modulus, which is equal to cross-sectional area divided by half the subperiosteal width (i.e. the maximum distance between the center of mass and the outer cortex) strengthens bone in bending [117]. A higher buckling ratio (outer radius of bone divided by the thickness of the cortical wall) on the other hand is associated with susceptibility of bone to buckling when placed under compression [117]. One year of CrM (0.1 grams/kg/day) combined with whole-body resistance training (3 days/week) (including loaded ankle dorsiflexion) improved bone area in the distal tibia (+17 vs. −1 mm^2^) and tibial shaft (0 vs. −5 mm^2^) (this potentially improves strength of bone under compression) in men and women (mean age ~58 years of age) compared to placebo and the same exercise program; [115]. While these results are statistically significant, the changes are comparable to the precision errors (i.e. % coefficient of variation) and below the least significant changes for tibial bone area [118]. Two years of CrM (0.14 grams/kg/day) combined with whole-body resistance training (3 days per week) and walking (6 days per week) in postmenopausal women (~59 years of age) increased section modulus at the femoral neck (0% vs. −4.4%) and femoral shaft (0% vs. −1%), and cortical thickness (+1.7% vs. −3.4%) at the femoral shaft, and reduced buckling ratio at both the femoral neck (+2.8% vs. +5.5%) and femoral shaft (−2.2% vs. 0%) compared to placebo and the same training program, potentially improving bone bending strength and strength of bone when under compression [113]. Again, caution should be used when interpreting these changes, as they might be smaller than changes necessary to prevent fracture. For example, when compared to postmenopausal women without hip fracture, those with hip fracture had 8.8% and 5.2% lower section modulus at the femoral neck and femoral shaft respectively, 12.8% lower cortical thickness at the femoral shaft, and 19.8% and 16.2% higher buckling ratios at the femoral neck and femoral shaft, respectively [119]. When assessed a year after this two-year program, there were no differences between creatine and placebo groups for actual number of fractures (i.e. four fractures in each group), but the study was probably underpowered (*n* = 237) to show differences between groups over this short time period. Longer study periods with greater numbers of participants would be needed to demonstrate whether CrM provides actual clinical benefit for preventing fracture.

## 6. Falls

CrM has potential to prevent falls through improvement in neural function, or improvement in muscle quality, both of which can affect motor ability and therefore reduce susceptibility of falling in older adults. Creatine crosses the blood-brain barrier and may be protective for brain health with aging [120]. In a model of aged mice, CrM tended to reduce reactive oxygen species in the brain, reduced “lipofuscin,” which is considered an aging pigment, upregulated genes associated with neuronal growth, neuroprotection, and learning, and tended to improve function on a locomotor task (*p* = 0.054) [121]. Improvement in locomotor function occurs in older adults supplementing with creatine. Two years of CrM (0.14 grams/kg/day) combined with resistance-training (3 days per week) and walking (6 days per week) improved walking speed (by close to 0.1 m/s) over 80 meters in postmenopausal women (compared to placebo and the same exercise program) [113], a change that is considered meaningful in clinical populations [122]. CrM also improves lower-leg muscle quality (assessed as muscle density) during one year of supplementation in men and women (age ~58 years of age) compared to placebo during 3 days per week resistance training [123]. This measure of lower-leg muscle density is a good independent predictor of reduced falling risk in community-dwelling older adults [105], and the change with CrM supplementation over placebo (1.4 mg/cm^3^) [123] is greater than the difference between older adults classified as fallers versus non-fallers (1.3 mg/cm^3^) [105]. As previously discussed, meta-analyses of CrM (compared to placebo) during resistance-training programs in older adults show that CrM is effective for improving functional ability (as determined by “sit to stand” performance) [93,95] which is a good predictor of reduced falling risk in older adults [124]. Despite this potential for CrM to prevent falls in older adults, only one study has assessed the effect of CrM on actual number of falls, finding that 2 years of CrM during a resistance-training and walking program in postmenopausal women had no effect on the number of falls compared to placebo and the same exercise program up to a year after the supplement and exercise intervention [113]. This study was most likely underpowered to adequately assess effectiveness of CrM on falls (*n* = 237 participants) and falls were subjectively reported, which may have led to error. Longer duration of supplementation with larger numbers of participants would be required to determine whether CrM provides clinically relevant reduction in number or severity of falls.

## 7. Frailty

Frailty is usually assessed by five parameters including weak muscular strength, slow walking speed, self-reported exhaustion, unintentional weight loss, and low physical activity level [125]. One is usually classified as “pre-frail” when possessing 1–2 of these characteristics and “frail” when three or more of these characteristics are present. Older adults classified as “frail” had slower PCr recovery kinetics (this is proportionate to mitochondrial function) in the tibialis anterior compared to those classified as “pre-frail” and those classified as “pre-frail” had slower kinetics compared to non-frail individuals after 30 seconds of isometric plantar flexor exercise [126]. This implies that performance might be improved with CrM since CrM would theoretically stimulate the reverse of the creatine kinase reaction where creatine would combine with ATP to speed resynthesis of PCr [127]. Studies in frail older adults engaged in resistance training however show no added benefit of CrM. Two studies combining 106 frail older adults randomized to CrM (~6 grams/day) or non-CrM groups during 14–16 weeks of resistance training showed that training was effective for enhancing grip strength, leg press and bench press strength, lean tissue mass and vastus lateralis muscle cross-sectional area, and functional tests such as timed up and go and chair sit-to-stands, with no differences between groups [128,129]. Resistance training is a powerful and effective stimulus for improving muscle mass, strength, and functional performance and large improvements with resistance training in a group of people who are very weak at baseline may obscure any small additional benefit from CrM. We therefore speculate that the potent effect of resistance-training in very deconditioned individuals obscures CrM’s smaller effect.

## 8. Creatine supplementation and age-related metabolic disorders and musculoskeletal conditions

Beyond its role in energy metabolism, creatine may also contribute to maintaining acid-base balance [130], improving inflammatory markers [131], and reducing oxidative stress [132], and improving inflammatory markers [131]. The diverse impacts of creatine on metabolism and cellular function provide the basis for its potential role in health and disease. This is especially relevant to aging, a process marked by a progressive decline in physiological integrity [133], where creatine’s pleiotropic effects could be of high therapeutic value.

For instance, metabolic disorders impair the body’s ability to process energy substrates (glucose, fatty acids, and amino acids), disrupting normal metabolism, often resulting in obesity (excess and dysfunction of the adipose tissue), hyperglycemia (high blood glucose levels), and/or dyslipidemia (abnormal blood lipids levels). If not properly addressed, these disturbances may contribute to organ dysfunction and systemic complications [134]. The underlying causes of metabolic disorders are typically multifactorial, ranging from congenital (e.g. genetic mutations) to lifestyle factors (e.g. poor dietary habits, physical inactivity), or a combination of these [135].

The global prevalence of obesity has increased rapidly over the last decades, reaching pandemic levels, with older adults showing the highest rates [136]. A relevant feature of this condition is the interaction between the immune system, particularly the recruitment of monocytes and macrophages, and adipose tissue, which triggers low-grade chronic inflammation [137]. This, in turn, may yield a vicious cycle of increased pro-inflammatory cytokine release, oxidative stress, and fat accumulation. Lifestyle interventions such as diet and exercise remain the most widely recommended strategies for managing obesity. Beyond its effects on inflammatory marker [131] and oxidative stress [132], emerging evidence from animal studies suggests that creatine may also influence adipocyte function and fat metabolism, contributing to increased energy expenditure and thermogenesis [138]. Although CrM alone does not appear to significantly reduce fat mass, a systematic review and meta-analysis of 609 participants aged ≥50 years found that CrM during resistance training caused a greater reduction in body fat percentage compared to placebo and resistance training [139]. While the majority of studies have lasted ≤16 weeks, given its additive effects on resistance training-induced adaptations, it is reasonable to speculate that the effects of CrM should be even more pronounced in the long-term.

Type 2 diabetes mellitus (T2DM) is a leading metabolic disorder characterized by persistent hyperglycemia (high blood glucose levels) resulting from impaired insulin secretion, reduced insulin sensitivity (insulin resistance), or both [140]. Insulin is a peptide hormone that helps regulate glucose levels by promoting glucose uptake in insulin-sensitive tissues. In skeletal muscle, the largest site for glucose disposal, insulin stimulates the translocation of glucose transporter type 4 (GLUT-4) to the cell membrane thereby promoting glucose entry [141]. Mechanistic research has identified several ways in which CrM could influence glycemic control (for a comprehensive review, see [142]), including: (i) enhanced pancreatic β-cell insulin secretion [143]; (ii) improved cellular hydration status, intracellular osmolarity, and osmosensing gene expression [144,145]; (iii) increased GLUT-4 content and translocation in an insulin-independent manner [146,147]; and, (iv) enhanced exercise-related effects on glucose uptake and insulin sensitivity [148]. Indeed, clinical evidence indicates that CrM, either alone or combined with exercise training, may improve glucose metabolism in both healthy older individuals and those with insulin resistance (e.g. patients with T2DM).

Previous studies in healthy young individuals have shown that CrM can prevent GLUT-4 protein expression declines caused by leg immobilization and even substantially increase it beyond baseline levels during rehabilitation training, while also improving oral glucose tolerance [149,150]. Although this still needs to be replicated in older adults, these findings are particularly relevant since this population faces a higher risk of immobilization and bedrest due to greater risk of falls and fractures. Gualano et al. [147] conducted a small-scale, double-blind, placebo-controlled trial to investigate the effects of CrM (5 grams/day for 12 weeks) combined with moderate-intensity aerobic and resistance training in individuals with T2DM [147]. Results showed that CrM led to greater reductions in glycemia and glycated hemoglobin (HbA1c), a central indicator of long-term glycemia control, while also improving glucose tolerance and GLUT-4 translocation. An ancillary analysis from this study found that HbA1c levels and increased GLUT-4 translocation were associated with increased AMP-activated protein kinase (AMPK) protein expression, a key cellular energy sensor and metabolic regulator [151]. These findings highlight the synergistic effect of combining CrM with exercise training to improve glucose metabolism, especially in clinical populations.

Since both glycemia and/or insulinemia are linked to blood lipoproteins levels [152,153], CrM supplementation may also have beneficial effects in other metabolic conditions, such as (e.g. obesity, dyslipidemia, and nonalcoholic fatty liver disease, metabolic syndrome), by helping improve the lipid profile. Furthermore, high creatine intake decreases the formation of SAMe, which reduces hepatic production of homocysteine, leading to a decreased synthesis and accrual of triglycerides and fat in the liver [154]. Preclinical studies using both *in- vitro* and rodent models have demonstrated that creatine can directly influence lipid metabolism by stimulating lipoprotein secretion and oxidation, thereby preventing lipid accumulation in the liver, and enhancing its metabolism improving overall lipid profile [155–158]. For instance, Marinello et al. [159] found that CrM attenuated liver fat accumulation and liver damage, preventing the progression of high-fat diet-induced nonalcoholic fatty liver disease in mice. Similarly, Deminice et al. [155] found that creatine supplementation prevented hepatic steatosis and lipid peroxidation in rats on a high-fat diet [156], as well as prevented increases in liver fat, cholesterol, triglycerides, and markers of inflammation and oxidative stress in rats fed a choline-deficient diet [155]. On the other hand, while a few human studies have reported improvements in lipid profiles with CrM [160,161], the majority have found no significant effects most have found no significant effect of CrM on the lipid profile [162–164]. However, it is important to note that the baseline lipoprotein concentrations in the study samples, mainly composed of healthy young individuals, were already within the normal range, which may help explain these inconsistent results. In contrast, using a sample of middle-aged adults with hypercholesterolemia, Earnest et al. [160] reported that CrM (20 grams/day for the first 5 days, followed by 10 grams/day for the remaining 51 days) reduced plasma total cholesterol, triacylglycerols, and very-low-density lipoprotein-C [160]. Although these results are promising, further research is needed in humans, particularly in older populations with clinical conditions.

Rheumatic conditions encompass a broad group of inflammatory and/or autoimmune diseases that primarily affect the musculoskeletal system, including skeletal muscles, joints, bones, and connective tissues. CrM has emerged as a promising non-pharmacological intervention for managing these conditions as it may enhance the benefits of exercise on muscle strength and function, even in older adults [165]. Neves et al. [166] demonstrated that CrM (20 grams/day for 7 days, followed by 5 grams/day for 11 weeks) enhanced the effects of resistance training in postmenopausal women with knee osteoarthritis, a condition characterized by pain, morning stiffness, and muscle weakness, all of which contribute to reduced functional capacity [166]. Compared to the placebo group, only those who supplemented with creatine showed significant improvements in lower-limb lean mass, physical function and stiffness. Similar results have been observed in individuals with rheumatic arthritis and fibromyalgia, with reports of increased muscle mass [167] and strength [168], respectively. Importantly, no adverse effects were reported in any study.

Overall, limited evidence seems to support the benefits of CrM as an adjuvant tool to help manage metabolic disorders and musculoskeletal conditions. However, research on older clinical populations remains scarce, with existing trials being mostly small-scale, short-term, and exploratory. Future research should focus on conducting larger, well-controlled randomized clinical trials with extended follow-up periods to better assess the long-term effects of CrM. Additionally, studies should investigate optimal dosing strategies for these purposes, either alone or in combination with other pharmacological and non-pharmacological interventions (e.g. exercise training), and the mechanisms underlying its effects in aging populations.

## 9. Creatine supplementation for neuromuscular disorders

Neuromuscular disorders are genetic or acquired disorders of the nerves, neuromuscular junction NMJ) or skeletal muscle that cause varying degrees of weakness, atrophy and/or exercise intolerance. Some of the more common disorders include; motor (spinal muscular atrophy (SMA), amyotrophic lateral sclerosis (ALS)) and motor-sensory (Charcot-Marie-Tooth (CMT)) neuropathies, NMJ disorders (myasthenia gravis and congenital myasthenic syndrome), and myopathies (dystrophy, congenital myopathy, inflammatory myopathy, metabolic myopathy). At the cellular level, there is evidence for similar final common pathways of oxidative stress, mitochondrial dysfunction, stem cell depletion, reduced protein synthesis, apoptosis, and inflammation [44]. In fact, most of these pathophysiological features are also seen in various tissues with aging [169]. CrM could have potential beneficial effects in neuromuscular disorders by increasing lean mass, muscle accretion, strength or endurance, lowering calcium or reactive oxygen species, activating stem cells, and/or reducing apoptosis [170,171]. Another potential benefit of CrM is the replacement of a deficiency state as seen in genetic, inflammatory [172], and mitochondrial [173], myopathies [174].

Corticosteroid drugs are used to treat certain neuromuscular disorders (i.e. myasthenia gravis, inflammatory myopathies, Duchenne muscular dystrophy (DMD)); however, these drugs can have negative effects on bone (short stature, osteoporosis) and muscle (type 2 fiber atrophy). Several research groups [175,176] have shown beneficial effects of CrM upon metrics of bone health in murine models. Two studies have shown lower markers of bone breakdown (N-telopeptides) in boys with DMD with CrM [177,178].

## 10. Neuropathies

Amyotrophic lateral sclerosis (ALS) is a degenerative disorder of the alpha motor neurons and corticospinal tracts due to a genetic (~15 %) or idiopathic etiology. Muscle atrophy/weakness leads to respiratory failure and wheelchair requirement over ~2–5 years. Treatment is supportive, although a small survival benefit is seen with a drug called Riluzole [179]. Interest in the use of CrM as a potential therapy came from a seminal paper showing a significant survival benefit in the G93A murine genetic ALS model [180], which was further replicated [181]. Unfortunately, these pre-clinical studies did not translate to significant benefits in human clinical trials [182], potentially due to the fact that the majority of nerves have dropped out by the time the diagnosis is made and the therapeutic window is very narrow at that time point.

Spinal muscular atrophy (SMA) is an autosomal recessive genetic motor neuron disorder due to bi-allelic mutations in the *SMN1* gene. The gene product, SMN1, is critical for the survival of alpha motor neurons *in utero* and in early post-natal life. Patients with SMA lose alpha motor neurons after birth and the severity and rapidity of the loss is inversely proportionate to the number of copies of a protective gene called *SMN2* [183]. Historically, most children with the more severe form, SMA1, die before 2 years of age; however, the advent of AAV9 based gene therapy [184], and new-born screening [185], have revolutionized therapy with most of our treated children walking by 18 months of age. In post-natal life, the importance of SMN1 drops rapidly [186], and the remaining nerves roughly dictate the residual weakness; however, a significant concern is that humans lose alpha motor neurons from early middle age till older age and this is accelerated in patients with prior motor neuron loss [187]. This loss is due to multiple final common pathways of neuronal loss [44]; amenable to exercise [188,189], and potentially, CrM patients with SMA who will now be surviving into adulthood represent an ideal group for future studies using CrM, exercise [190], and other mitochondrial/anti-oxidant [191] strategies.

Charcot-Marie-Tooth (CMT) are a group of genetic neuropathies that affect both the motor and sensory nerves. They lead to progressive muscle atrophy/weakness in the distal muscles of the feet and eventually hands. Two studies involving adults with CMT have produced equivocal findings, potentially by being underpowered (i.e. type II error). One study failed to observe greater strength and functionality adaptations from CrM (5 grams/day) above exercise training alone [192]; however, another study showed that CrM enhanced MHCIIa content in CMT patients during resistance training and this correlated with better function [193].

## 11. Neuromuscular junction disorders

Myasthenia gravis (MG) is an autoimmune disorder due to antibodies against proteins at the neuromuscular junction (mainly anti-acetylcholine receptor antibodies), that results mainly in fatiguable ocular and/or bulbar weakness, with some patients also getting muscle weakness and respiratory failure. Because the mainstay of therapy is the use of corticosteroid medications, there is potential for CrM mainly as a countermeasure to the deleterious effects of steroids as mentioned above. A case report found improvements in lean mass and strength with resistance exercise and CrM in a man with MG [194]. Congenital myasthenic syndromes are a group of genetic disorders manifesting with oculo-bulbar ± limb weakness and due to mutations in genes encoding for proteins involved in the neuromuscular junction. To date, there are no studies with CrM in this group but it could be a potential therapeutic adjunct.

## 12. Muscular dystrophies

Muscular dystrophies are genetic disorders affecting proteins involved in the structure or function of skeletal muscle. These result in progressive and mainly proximal muscle weakness with cardiac and respiratory manifestations seen in some (i.e. cardiomyopathy in DMD, conduction block in myotonic MD). The most common form of muscular dystrophy is Duchenne (DMD) affecting ~1/3,500 live male births with a less common and milder form called Becker MD (BMD), due to complete and partial absence of dystrophin protein, respectively. Corticosteroids represent the only approved therapy for DMD in spite of the significant side effects [195]. Pre-clinical studies have shown pathological and functional benefits from CrM supplementation in murine models of DMD [196–199], and also in a murine model of fascio-scapulo-humeral dystrophy (FSHD) [200]. Clinical studies with CrM have been largely positive in DMD/BMD [177,178,201], equivocal in myotonic dystrophy (type I and II) [202–204], and positive in mixed myopathy populations [201,205].

## 13. Inflammatory myopathies

Inflammatory myopathies are a group of disorders characterized by primary muscle inflammation (mainly T or B cells). Most IMs show proximal weakness (dermatomyositis, polymyositis, anti-synthetase, overlap myositis, autoimmune necrotizing myopathy); whereas, sporadic inclusion body myositis (sIBM) shows a characteristic pattern of deep finger flexion and quadriceps weakness and is 3 times more common in older men [206]. All of the IMs (except sIBM) are treated with corticosteroids and this alone represents a rationale for the use of CrM; however, there is lower total creatine and PCr content in muscle of IM patients [171,173] and the potential to replace a deficiency further strengthens the rationale for therapy.

A clinical trial in patients with polymyositis or dermatomyositis (all taking corticosteroids) and performing home based exercise showed greater efficacy in those taking CrM in functional outcomes and muscle PCr [207]. A study in juvenile dermatomyositis (*n* = 15; 7–21 years of age) found that CrM (0.1 grams/kg/day) was well tolerated but did not increase strength or muscle PCr content after 12 weeks [208]. In contrast, a larger study in 29 patients (completers) with dermatomyositis or polymyositis (all on immunosuppressants and/or corticosteroids) performing home based exercise showed increased PCr content and better symptom reduction for those on CrM (20 grams/day for 8 days followed by >3 grams/day for 6 months) vs. placebo [207]. Further, CrM improved strength outcomes (by 11%) in IM patients (including sIBM) 81) [205]. Given the known benefits of exercise training in sIBM to improve strength or attenuate decline [209–212], and the synergy seen with CrM and resistance training in older adults [213,214], it would be ideal for this combination to be evaluated in sIBM.

## 14. Metabolic myopathies

Metabolic myopathies are genetic disorders affecting the pathways of intermediary metabolism including disorders of; glycolysis (i.e. Tarui disease), glycogenolysis (i.e. McArdle disease), fatty acid oxidation (i.e. CPT2 deficiency), and mitochondria [215]. Anaerobic glycogenolysis/glycolysis and phosphocreatine are the main energy sources during higher intensity exercise and in the rest to exercise transition. Consequently, with either glycogenolytic or glycolytic defects, CrM should be ideal to increase PCr concentration and provide an anaerobic energy buffer. One randomized, double-blind study reported greater muscle force and PCr utilization in McArdle disease patients (*N* = 9) after five weeks of CrM (0.15 grams/kg/day for 5 days followed by >0.06 grams/kg/day for 4.5 weeks) [216]. In contrast, 5 weeks of CrM (0.15 grams/kg/day for 5 weeks) actually led to more muscle pain/cramps and daily activity [217], potentially due to the fact that the higher dose likely impaired their disease up-regulated phosphofructokinase (PFK) activity [218,219], and/or the lack of proton formation in McArdle disease would impair the forward flux of the PCr reaction (PCr + ADP + H^+^ → ATP + Cr). To date the efficacy of CrM for treating glycolytic defects is unknown.

Fatty acid oxidation defects are genetic disorders that affect either β-oxidation or long-chain fatty acid transport into the mitochondria. They present with myalgia and pigmenturia (myoglobin) with fasting, super-imposed illness and/or prolonged exercise and patients with β-oxidation defects often have muscle atrophy/weakness and/or cardiomyopathy. There appears to only be one case report of a patient with long chain β-OH acyl CoA-dehydrogenase (LCHAD) deficiency who lost the ability to walk at 11 y and was able to walk within a month of starting CrM (0.13 gram/kg/d) and was able to still walk, cycle and climb stairs at 16 years of age still on therapy [220].

The final common pathway for aerobic energy utilization is in the mitochondria. Mitochondrial myopathies are due to genetic mutations in either the mitochondrial (mtDNA) or nuclear (nDNA) DNA encoding for components of mitochondrial structure/function. The myopathy can present with fixed weakness and/or exercise intolerance and/or rhabdomyolysis [215]. In theory, patients with mitochondrial myopathies could benefit by enhancing the creatine-phosphocreatine shuttle [221], attenuating apoptosis [222], reducing paracrystalline inclusions [223], replacing the PCR/Cr depletion state in muscle [174], and/or provision of an alternative energy source to lower lactate. There is some evidence of higher strength and lean mass in MELAS (mitochondrial encephalomyopathy, lactic acidosis and stroke-like episodes) patients [224]. Two other small (*n* = 4 each) case series found improvements in endurance exercise metrics with varied CrM doses (0.1 → 0.35 g/kg/d) and duration (up to three years) in patients with a variety of different mitochondrial myopathies [225,226]. Chronic Progressive External Ophthalmoplegia (CPEO) is usually a sporadic disorder seen in older adults with ophthalmoplegia, ptosis, hypoacusis, dysphagia and muscle weakness [227]. One prospective clinical trial found no significant benefits (strength or neuromuscular symptom score) in 16 patients with CPEO after a month of high dose CrM [228]. Another clinical trial failed to show an increase in muscle PCr/ATP or strength in 15 patients with CPEO after six weeks of CrM (0.15 grams/kg/day) [227]. Both studies found trends toward higher strength outcomes and suggested that further research was required [229].

In summary, the data to date does not support a role for CrM in ALS, CPEO, and there is too little data in peripheral nerve or neuromuscular junction disorders to render a conclusion. The literature remains generally supportive of the conclusions of the 2013 Cochrane review showing that CrM (~0.1 grams/kg/day) improves strength (~8.5 %) and lean mass (~0.63 kg) in patients with MD [230]; however, this does not translate to myotonic MD patients. It is very likely that the full potential of CrM will be only apparent when combined with exercise therapy as seen in the aging population [213,214], and inflammatory myopathies [207]. In addition, it is likely that the potential benefits of CrM will be best realized when it is a part of a multi-ingredient supplement strategy that targets the final common pathways of muscle pathology in patients with myopathies [44]. Indeed, the combination of CrM, alpha-lipoic acid, vitamin E and coenzyme Q10 lowered lactate and markers of oxidative stress in patients with mitochondrial myopathy [191], yet there were no benefits from very high dose of coenzyme Q10 in a similar but larger cohort of patients [231]. The addition of CrM to vitamin D, calcium and omega-3 fish oil to high quality protein has been shown to enhance strength and function during resistance exercise training in older adults [232,233].

## 15. Creatine supplementation and the aging brain

With the global rise in the aging population, cognitive decline has become increasingly prevalent. The United Nations projects that, by 2050, the global number of individuals aged ≥ 65 will double to 1.6 billion people [234]. This rapid increase in older adults will not only increase the prevalence of disease and disability but will also highlight concerns about impairments in cerebral functioning.

Deficits in executive function (e.g. decision making and working memory), concentration, and reaction time are hallmark characteristics of cognitive impairment in older adults. These impairments range from mild deficits, which may not be clinically detectable, to diseases like Dementia and Alzheimer’s Disease (AD). There are many different mechanisms associated with age-related cognitive impairment, with examples including vascular conditions and neuronal degeneration [235]. Age-related cognitive decline can impair an individual’s ability to perform activities of daily living, increasing the risk of falls, dementia, and ultimately, mortality. These impairments not only diminish quality of life but also threaten independence and autonomy. As cognitive decline progresses, many older adults require caregiver support and have a greater reliance on healthcare services, further contributing to increased mortality risk.

Creatine plays a vital role in the (PCr) energy system, acting as an ATP buffer in tissues that have significant and fluctuating energy demands, such as the brain, to sustain neuronal function [236]. Although only comprising approximately 2% of total body mass, the brain is a highly metabolically active organ, accounting for about 20% of resting energy consumption. Interestingly, the rate of brain metabolism remains relatively constant despite varying challenges posed by cognitive and motor tasks [237] thereby suggesting a great demand for high-energy compounds such as PCr in the brain. In fact, it has been shown that genetic creatine deficiencies (e.g. GAMT deficiency) or low levels of creatine in the brain lead to clear developmental delays, extrapyramidal movement disorders and seizures [238]. To sustain a consistent creatine supply, the brain depends on multiple sources, including dietary intake from creatine-rich foods or supplementation and endogenous synthesis in the liver and brain cells [239]. However, the relative contribution of each source remains unclear and is likely influenced by dietary creatine availability, precursor molecule intake, and the efficiency of creatine transport and synthesis mechanisms both within and outside the central nervous system (CNS).

Although skeletal muscle has an outstanding capacity to uptake creatine, that is not the case in the brain. The brain’s ability to absorb creatine is limited by the blood-brain barrier (BBB) and a lack of SLC6A8 transporters in the astrocyte feet lining the microcapillary endothelial cells [240]. Despite these limitations, some of the dietary creatine and creatine synthesized in the liver enters the brain via the SLC6A8 Cr transporter (CT1), which is expressed in the microcapillaries of the BBB, neurons, and oligodendrocytes [241]. Evidence suggests that CrM has the potential to elevate brain creatine and PCr levels, which is associated with increased brain bioenergetics and improved cognitive performance [242]. These positive metabolic effects may promote better cognitive functioning during periods of increased ATP demand (sleep deprivation, hypoxia or mental fatigue) or for those who may be reduced brain creatine levels (elderly or those with pathophysiological states such as creatine deficiency syndrome) [240,243–246]. In addition to bioenergetics, creatine has been reported to have anti-apoptotic, anti-excitotoxic, and anti-oxidative properties *in vivo* and *in vitro* [11,132,247]. This ensures protection against cell death and promotion of survival and differentiation of neurons [248]. Recently, Zhu et al. [249] reported that mice supplemented with 3% creatine showed reduced cognitive impairment, blunted oxidative stress, improved and hippocampal structural plasticity in response to injections designed to initiate cognitive decline (D-galactose). In addition, the mice who received CrM had a 14.3% increase in CK-BB expression and increased CK-BB activity. These results suggest that CrM may act as a neuroprotective substance in mice by preventing or delaying age-related cognitive decline.

Like skeletal muscle, brain creatine stores are thought to decrease with age, potentially contributing to reduced brain activity and disease [250,251]. Aging is strongly associated with structural and functional brain deterioration, diminished mitochondrial oxygen consumption, and decreased ATP synthesis [252]. Age-related changes in neural activity and brain organization particularly affect the prefrontal cortex (PFC). The PFC is a region highly active during tasks of executive function [253] and neural activity in this area has been shown to become less localized with age over time [254]. Interestingly, older adults who engage both hemispheres of the PFC tend to perform cognitive tasks more effectively than those who show asymmetrical activation [255], suggesting that maintaining energy availability is crucial for cognitive performance. Thus, the role of creatine in energy metabolism highlights the importance of adequate energy availability during mentally stimulating tasks. Given the widespread prevalence of age-related cognitive, structural, and metabolic decline, the need to investigate CrM as a tool to improve overall brain health remains an important consideration. One important thing to note is that there is substantial inter-individual variation in brain creatine content, due to the variable nature of physiological changes associated with aging. Consequently, chronological age alone should not determine the need for supplementation – a more precise approach would involve directly measuring brain creatine levels in individuals to assess supplementation needs.

Only a limited number of studies have explored the effects of CrM on cognitive function in older adults. McMorris et al. [245] reported significant improvements in working memory and long-term memory in healthy older adults following 7 days of CrM. Similarly, Alves and colleagues [168] found that 16 weeks of CrM improved incidental memory in older adults with fibromyalgia. These studies are supported by a recent systematic review and meta-analysis that reports CrM results in improved memory measures in healthy older adults (66–76 years of age) compared to placebo across randomized controlled trials (RCTs) [256]. These findings are supported by a second systematic review and meta-analysis that reports improvements in memory (but not attention or overall executive function) in healthy and diseased adults (e.g. fibromyalgia and mild cognitive impairment associated with Parkinson Disease) as a result of CrM [257]. In contrast, another study by Alves et al. [258] found no significant improvements across all measured cognitive variables in postmenopausal females following 24 weeks of CrM. A key difference between the aforementioned RCTs was the dosage and duration of CrM supplementation. Specifically, McMorris et al. [245] utilized 4 × 5 grams/day for 7 days, whereas Alves et al. [168] supplemented with 4 × 5 grams/day for 5 days, followed by 1 × 5grams/day for 107 days and Alves et al. [258] administered 4 × 5 grams/day for 5 days, followed by 1 × 5 grams/day for 163 days. Based on dosing duration, one might expect the longer supplementation protocol in Alves et al. [258] to produce greater cognitive benefits. However, age and health differences between study participants may have also influenced outcomes, as McMorris et al.’s sample had a mean age of 76 years (SD = 9), whereas Alves et al. [168] recruited adults (~49 years of age) with fibromyalgia and Alves et al. [258] included postmenopausal participants ranging from 60 to 80 years old (mean age = 67 years).

Only two studies have measured changes in brain creatine content in older adults. In the most recent study, Smith et al. [259] showed that CrM (20 g/day) for 8 weeks significantly increased brain total creatine levels by 11% (*p* < 0.001) and measures of cognition and memory in 20 older adults (73 years of age) diagnosed with Alzheimer’s Disease. In contrast, Solis et al. [260] failed to observe a significant increase in brain creatine levels following 7 days of CrM (0.3 grams/kg/day) in older adults [260], suggesting that longer supplementation periods or higher doses may be required to achieve cognitive benefits in aging and/or clinical populations. These variations in participant age, health status and treatment protocols highlight the need for future research to control for total brain creatine content and other individual differences when assessing the cognitive benefits of CrM in older adults.

Interestingly, recent studies have explored the potential synergistic effects of CrM with antidepressant medications. An open-label pilot study demonstrated that the combination of 5-hydroxytryptophan (5-HTP) and CrM (5 grams/day) twice daily for 8 weeks provided potential benefits for adult women with serotonin-norepinephrine reuptake inhibitor (SNRI)-resistant depression [261]. Most recently, Sherpa et al. [262] showed that CrM (5 grams/day) could augment the antidepressant effects of cognitive behavioral therapy (CBT). While the mechanisms explaining the possible antidepressant effects of creatine remain unknown, it has been recently proposed that creatine acts as a neuromodulator and neurotransmitter, acting on the D_1_ and D_2_ dopamine receptors, serotonergic 5-HT _1A_ receptors, α_1_-adrenoceptors and adenosine A_1_ and A_2a_ receptors [263].

Beyond oral supplementation, previous research has examined the impact of dietary creatine (i.e. from food sources) in older adults. Machado et al. [264] found that older women who consumed high levels of creatine from their diet ( >1 grams/day, approximately 200–250 grams of red meat or fish) performed better on cognitive tasks involving attention and select inhibition in comparison to females who consumed <1 grams/day of creatine from their diet. In addition, an innovative pilot study by Chen et al. [265] investigated the intravenous administration of PCr or ATP in combination with fluoxetine, demonstrating a novel strategy to enhance antidepressant efficacy. While preliminary, this approach suggests a promising direction for future research involving CrM in psychiatric treatment. Despite these positive findings, more research is needed to elucidate the exact effect of dietary or supplementary creatine in maintaining brain creatine stores and improving both brain and overall health.

One possible explanation for the inconsistencies observed in much of the CrM cognitive functioning research is the limited increase in brain CrM levels in older adults, even following standard supplementation protocols. For example, Solis et al. [260] reported no significant increase in brain CrM using ^31^P-MRS following 7 days of supplementation with 0.3 grams/kg/day in older adults. This raises an important question about the true potential of CrM to enhance cognitive functioning in this population. It may be that higher doses, longer supplementation durations, or novel delivery forms (e.g. combination with medications, CrM ethyl ester or hydrochloride, or chewable CrM gummies) are necessary to overcome BBB limitations and increase neural uptake. While direct measurement or brain CrM would provide valuable insight, it is currently impractical to measure this on a large scale. Therefore, perhaps future research may consider stratifying participants by baseline cognitive performance scores or using indirect markers of brain bioenergetics (e.g. fNIRS/EEG) to tailor CrM protocols more effectively.

In summary, evidence suggests that adequate creatine intake whether through diet or supplementation, may support memory and cognitive function in older adults, offering a promising avenue for brain health. Further research is needed to determine optimal dosing strategies for increasing brain creatine levels in older adults and the underlying mechanisms involved. In addition, future research should investigate the potential synergistic effects of CrM and current treatments for physical and mental health disorders. Overall, CrM in older adults shows promise in enhancing cognitive function, supporting brain energy metabolism, and potentially serving as an adjunct therapy for mood disorders. However, long-term, large-scale trials are needed to establish its role as a clinical intervention for cognitive aging and neurodegenerative conditions such as Dementia and AD.

## 16. Safety of creatine supplementation in older adults

The safety of CrM has been extensively documented across diverse populations in the scientific literature (see Kreider et al., [1], for a comprehensive review). Acknowledged as a Generally Recognized As Safe (GRAS) food compound, CrM has also undergone rigorous safety evaluations by regulatory authorities, including the U.S. Food and Drug Administration, which has confirmed its safety when used as intended. However, there has been relatively limited scientific focus on the safety of CrM in older populations. This represents a critical gap, especially considering the age-related changes in pharmacokinetics and pharmacodynamics that could alter the body’s response to many substances, potentially increasing the risk of toxicity or adverse reactions if doses are not appropriately adjusted [266]. Preliminary studies have demonstrated differential physiological responses to CrM between young and older individuals [260,267], underscoring the need for an age-sensitive assessment of creatine safety in older adults. Furthermore, there is a notable lack of research on how CrM may interact with common medications or comorbidities in this vulnerable population. This gap highlights the necessity of investigating potential drug-nutrient interactions and the influence of age-related physiological changes on creatine metabolism and safety.

Available data on CrM safety in older populations are typically derived as secondary outcomes from efficacy studies in which creatine is administered alongside resistance training and/or other nutrients. This approach limits the understanding of creatine pharmacovigilance on its own, particularly in vulnerable populations. The variability in treatment durations and dosages further complicates the assessment of safety. Additionally, the safety indicators used in these studies have been relatively limited, primarily focusing on subjectively reported side effects, traditional clinical enzymes, blood chemistry components, and surrogate markers of organ function. According to these studies, CrM does not appear to induce significant clinician- or patient-reported side effects, nor does it impair typical clinical biomarkers of safety (for a detailed review, see [268]. Subjectively reported side effects include gastrointestinal disturbances [111,214–271], muscle discomfort [111,271,272], overuse trauma [271,273], chest cold [271], and increased sweating/hot flashes [274]. These effects were generally minor, transient, and infrequent. Whether muscle-related symptoms are attributable to CrM or to myofibrillar damage induced by exercise remains unclear in this population. Recently, a comprehensive safety analysis was performed and found that in older adults with 32 published studies and 1232 participants, there was no significant differences in any evaluated side effects [275].

Several studies have demonstrated increased serum and/or urinary creatinine levels following CrM in a dose-dependent manner in older populations [267,276,277]. This is likely due to enhanced creatine utilization/metabolism rather than kidney dysfunction. It is due to a higher rate of creatine > creatinine conversion in muscle and muscle mass and creatinine are positively correlated). A rare creatine-only long-term study involving older adults with Parkinson disease found that supplementation with 4 grams of creatine per day resulted in an increase in serum creatinine, but no other kidney function markers were significantly affected over a two-year period [120]. This suggests a negligible risk of kidney dysfunction associated with low-dose CrM. Interestingly, CrM has been shown to interact with several pharmaceutical agents, including pemetrexed, entecavir, cimetidine, trimethoprim, and probenecid, as indexed in DDInter, a comprehensive database dedicated to drug interactions. The administration of CrM to older adult patients taking these medications may result in elevated serum levels of both the drugs and creatinine, raising potential safety concerns regarding the use of CrM in all patients taking these medications. This underscores the need for careful monitoring and further research to assess the safety and potential interactions between CrM and specific drugs (also food compounds) in this population.

Overall, CrM is generally safe for older adult populations when co-administered with exercise training or in rare creatine-only trials. However, there is an urgent need for older adult focused safety studies conducted in accordance with Good Pharmacovigilance Practices to properly evaluate clinical and laboratory manifestations of possible adverse events of CrM. Such studies should consider demographic characteristics, exposure duration, time from initiation of creatine intake to adverse event occurrence, doses used (including labeled doses and overdoses), concomitant medications, the presence of co-morbid conditions (particularly those known to increase the risk of adverse events, such as hepatic or renal impairments), specific creatine formulations, and changes in event reporting rates over the product lifecycle. Considering the numerous benefits of CrM for older adults, both in conjunction with and independent of exercise interventions [103,278,279], the availability of robust, evidence-based safety data would significantly enhance its potential for widespread application within this rapidly expanding demographic.

## 17. Importance of food-sourced creatine for healthy aging

Animal-based foods are a primary dietary source of exogenous creatine for most individuals, with an omnivorous diet – comprising fish, poultry and meat – estimated to provide approximately 50% of daily creatine requirements [6]. Despite this, dietary reference intakes for creatine remain undefined, particularly for older adults, leaving populations without specific nutritional recommendations for this conditionally essential nutrient [280–282]. Emerging evidence suggests that dietary creatine needs may vary based on factors such as dietary habits, body size, muscle mass, and physical activity levels [2]. Given that aging compromises these factors, older adults may require additional creatine to maintain optimal levels. Epidemiological data indicates that lower dietary creatine intake in older adults is associated with increased risks of various health conditions affecting energy-demanding organs. For example, older adults consuming <0.95 grams of creatine per day were shown to perform worse on cognitive functioning tests compared to peers with higher creatine intake [24]. Additionally, suboptimal dietary creatine intake ( <1 grams/day) was linked to a 2.62-fold higher risk of angina pectoris and a 2.59-fold higher risk of liver conditions, even after adjusting for demographic and nutritional variables [25]. These findings suggest that dietary creatine may play a protective role in preserving cognitive performance and supporting cardiovascular, liver, and potentially muscular health in aging populations. Addressing this nutritional gap could be a crucial public health initiative to promote health and functionality during aging. Strategies to increase creatine intake include promoting the consumption of creatine-rich foods, recommending CrM, or utilizing creatine-fortified foods [283]. While supplementation may effectively maintain creatine levels, dietary sources remain particularly important for individuals who prefer whole-food solutions or choose not to use supplements.

Despite increasing interest in the role of food-sourced creatine for older adults, significant knowledge gaps remain regarding its use and efficacy in this population. Notably, there are no specific dietary guidelines for creatine intake tailored to older adult populations, despite their unique metabolic and muscle maintenance needs compared to younger individuals [284]. Limited data exist on the typical dietary intake of creatine among older adults, particularly across different cultural and dietary contexts. For example, many older individuals reduce meat consumption [285], the primary dietary source of creatine, potentially leading to suboptimal intake. Furthermore, the absorption and utilization of creatine from food sources in older adults are poorly understood, especially given age-related changes in digestion and metabolism [286]. The comparative efficacy of creatine from dietary sources versus supplementation remains unclear, particularly in supporting muscle mass, cognitive function, and other health outcomes. Additionally, the impact of biological aging on the metabolism and storage of dietary creatine, particularly in the context of reduced muscle mass, is insufficiently characterized. The potential interactions between dietary creatine and common conditions or medications in older populations, such as diabetes or statin use, are also understudied. Research often overlooks subgroups like frail older adults, those with sarcopenia, or vegetarians, who may have distinct creatine needs and intake patterns. Furthermore, environmental and ethical concerns associated with creatine-rich foods such as red meat highlight the need for exploring alternative sources [287], such as lab-grown creatine or fortified products, which remain largely unexplored. Addressing these gaps through targeted research is essential to elucidate the role of food-sourced creatine in promoting healthy aging, thereby informing dietary guidelines and strategies for improving health outcomes in older adults.

## 18. Summary

CrM is safe and has emerged as a promising strategy to support healthy aging, particularly when combined with exercise training, by increasing lean body mass, regional muscle size, strength and functional ability in older adult populations. There is also evidence that CrM increases bone area and bone strength. Subsequently, CrM and exercise training should be considered in the treatment regimen for those diagnosed with age-related sarcopenia and osteoporosis. Furthermore, preliminary research suggests potential benefits from CrM on measures of glucose kinetics and cognition and memory in healthy older adults and those diagnosed with Alzheimer’s Disease. Finally, CrM has widespread application for those with various neuromuscular disorders.
